# Dysregulation of Subcutaneous White Adipose Tissue Inflammatory Environment Modelling in Non-Insulin Resistant Obesity and Responses to Omega-3 Fatty Acids – A Double Blind, Randomised Clinical Trial

**DOI:** 10.3389/fimmu.2022.922654

**Published:** 2022-07-25

**Authors:** Helena L. Fisk, Caroline E. Childs, Elizabeth A. Miles, Robert Ayres, Paul S. Noakes, Carolina Paras-Chavez, Elie Antoun, Karen A. Lillycrop, Philip C. Calder

**Affiliations:** ^1^ Faculty of Medicine, University of Southampton, Southampton, United Kingdom; ^2^ School of Medicine, The University of Notre Dame Australia, Freemantle, WA, Australia; ^3^ Faculty of Environmental and Life Sciences, University of Southampton, Southampton, United Kingdom; ^4^ National Institute for Health and Care Research (NIHR) Southampton Biomedical Research Centre, University Hospital Southampton National Health Service (NHS) Foundation Trust and University of Southampton, Southampton, United Kingdom

**Keywords:** obesity, LC n-3 PUFA, adipose tissue, inflammation, tissue remodelling, Wnt signalling

## Abstract

**Background:**

Obesity is associated with enhanced lipid accumulation and the expansion of adipose tissue accompanied by hypoxia and inflammatory signalling. Investigation in human subcutaneous white adipose tissue (scWAT) in people living with obesity in which metabolic complications such as insulin resistance are yet to manifest is limited, and the mechanisms by which these processes are dysregulated are not well elucidated. Long chain omega-3 polyunsaturated fatty acids (LC n-3 PUFAs) have been shown to modulate the expression of genes associated with lipid accumulation and collagen deposition and reduce the number of inflammatory macrophages in adipose tissue from individuals with insulin resistance. Therefore, these lipids may have positive actions on obesity associated scWAT hypertrophy and inflammation.

**Methods:**

To evaluate obesity-associated tissue remodelling and responses to LC n-3 PUFAs, abdominal scWAT biopsies were collected from normal weight individuals and those living with obesity prior to and following 12-week intervention with marine LC n-3 PUFAs (1.1 g EPA + 0.8 g DHA daily). RNA sequencing, qRT-PCR, and histochemical staining were used to assess remodelling- and inflammatory-associated gene expression, tissue morphology and macrophage infiltration.

**Results:**

Obesity was associated with scWAT hypertrophy (*P* < 0.001), hypoxia, remodelling, and inflammatory macrophage infiltration (*P* = 0.023). Furthermore, we highlight the novel dysregulation of Wnt signalling in scWAT in non-insulin resistant obesity. LC n-3 PUFAs beneficially modulated the scWAT environment through downregulating the expression of genes associated with inflammatory and remodelling pathways (*P <*0.001), but there were altered outcomes in individuals living with obesity in comparison to normal weight individuals.

**Conclusion:**

Our data identify dysregulation of Wnt signalling, hypoxia, and hypertrophy, and enhanced macrophage infiltration in scWAT in non-insulin resistant obesity. LC n-3 PUFAs modulate some of these processes, especially in normal weight individuals which may be preventative and limit the development of restrictive and inflammatory scWAT in the development of obesity. We conclude that a higher dose or longer duration of LC n-3 PUFA intervention may be needed to reduce obesity-associated scWAT inflammation and promote tissue homeostasis.

**Clinical Trial Registration:**

www.isrctn.com, identifier ISRCTN96712688.

## Introduction

Obesity is characterised by an increase in adipose tissue mass and is accompanied by a state of chronic low-grade inflammation in which physiological functions of adipose tissue and whole-body homeostasis are dysregulated (1). Consequently, obesity and the expansion of white adipose tissue (WAT) are strongly associated with comorbidities such as insulin resistance and type 2 diabetes (2–5). WAT expansion, reorganisation, and associated inflammation link the pathophysiology of obesity with metabolic complications. The expansion of pre-existing adipocytes (hypertrophy) and reorganisation of the tissue environment are accompanied by reduced expandability of restricted adipocytes, distancing them from the vasculature resulting in regions of hypoxia (6–9), immune cell infiltration, and inflammatory signalling (10–14).

The expansion of WAT is under tight regulation of transcription factors, gene expression, and many inflammatory signalling mediators such as cytokines, which have a role in lipogenesis and lipolysis, and therefore energy regulation, as well as in the remodelling of the surrounding microenvironment (3, 15). Adipogenesis and WAT expansion are nutritionally regulated under the control of Wnt/β-catenin signalling (16). Wnt and dishevelled binding antagonist of beta catenin (DACT, alias DAPPER) ligand expression are differentially regulated in response to nutrient surplus, consequently upregulating the expression of adipogenic factors to accommodate the need for enhanced triglyceride (TG) storage (16). During WAT expansion, there is downregulation of Wnt expression, often observed in conjunction with upregulated expression of DACT genes (16, 17); however, regulation of this pathway in WAT in human obesity is not described (18). Furthermore, in adipocytes, the canonical Wnt/β-catenin pathway has been shown to regulate *de novo* lipogenesis and fatty acid monounsaturation (19).

As WAT expands and regions of the tissue become hypoxic, inflammatory signalling promotes the reorganisation of the tissue environment to reconnect with the vasculature. The presence and role of hypoxia in human obesity is not well described as reviewed by Ruiz-Odeja (20) but stabilisation of hypoxia inducible factor-1α (HIF-1α) in hypoxia stimulates the expression of a range of genes involved in angiogenesis, glycolysis, and erythropoiesis (7, 11, 20–23). Hypoxia promotes immune cell recruitment and elicits a fibrotic response by inducing the expression of many extracellular matrix (ECM) components such as collagens (11, 24). Examination of the WAT transcriptome has revealed dysregulated expression of many ECM components in obesity (25, 26). However, evaluation of changes to WAT morphology in human obesity is limited and inconsistent as reviewed by DeBari and Abbott (27).

Eicosapentaenoic acid (EPA) and docosahexaenoic acid (DHA) are the two most bioactive long chain omega-3 polyunsaturated fatty acids (LC n-3 PUFAs) and have been widely investigated for their anti-inflammatory actions. In addition to potentially influencing inflammatory mediator signalling, EPA and DHA elicit their actions *via* altering the activity of transcription factors to modulate inflammation and other processes in WAT (28, 29). We previously reported the modulation of the scWAT transcriptome in human obesity by EPA and DHA resulting in downregulation of genes involved in immune and inflammatory responses (30). Furthermore, EPA and DHA have been reported to regulate the expression of genes associated with lipid accumulation in WAT (31, 32), decrease hepatic fibrosis accompanying metabolic complication through decreasing expression of collagen-associated genes and the presence of collagen fibres (33, 34), and impair collagen reorganisation in wound healing in mice (35). Therefore, EPA and DHA may have the potential to modulate scWAT expansion and remodelling; however, the effects of LC n-3 PUFAs on these outcomes in scWAT in human obesity are not described.

In this study, we combine whole tissue transcriptome profiling, investigation of tissue morphology and macrophage infiltration, and inflammatory marker analysis to describe obesity-associated expansion and remodelling of scWAT, responses to chronic LC n-3 PUFA intervention, and possible mechanisms by which these occur.

## Material and Methods

50 healthy normal weight (BMI 18.5 to 25 kg/m^2^) and 50 healthy obese (BMI 30 to 40 kg/m^2^, waist circumference ≥ 94 cm males and ≥ 80 cm females) individuals aged 18-65 years who were able to provide written informed consent were recruited into a double-blind placebo (comparator oil) controlled trial. Exclusion criteria included: being outside the defined age or BMI and waist circumference categories, having diagnosed metabolic disease (e.g. diabetes, cardiovascular disease) or chronic gastrointestinal problems (e.g. inflammatory bowel disease, celiac disease, and cancer), the use of prescribed medicine to control inflammation, blood lipids or blood pressure, consumption of more than one portion of oily fish per week (140 g cooked), use of fish oil or other oil supplements, being pregnant or planning to become pregnant during the study period, and participation in another clinical trial.

### Study Design

**Figure 1:**
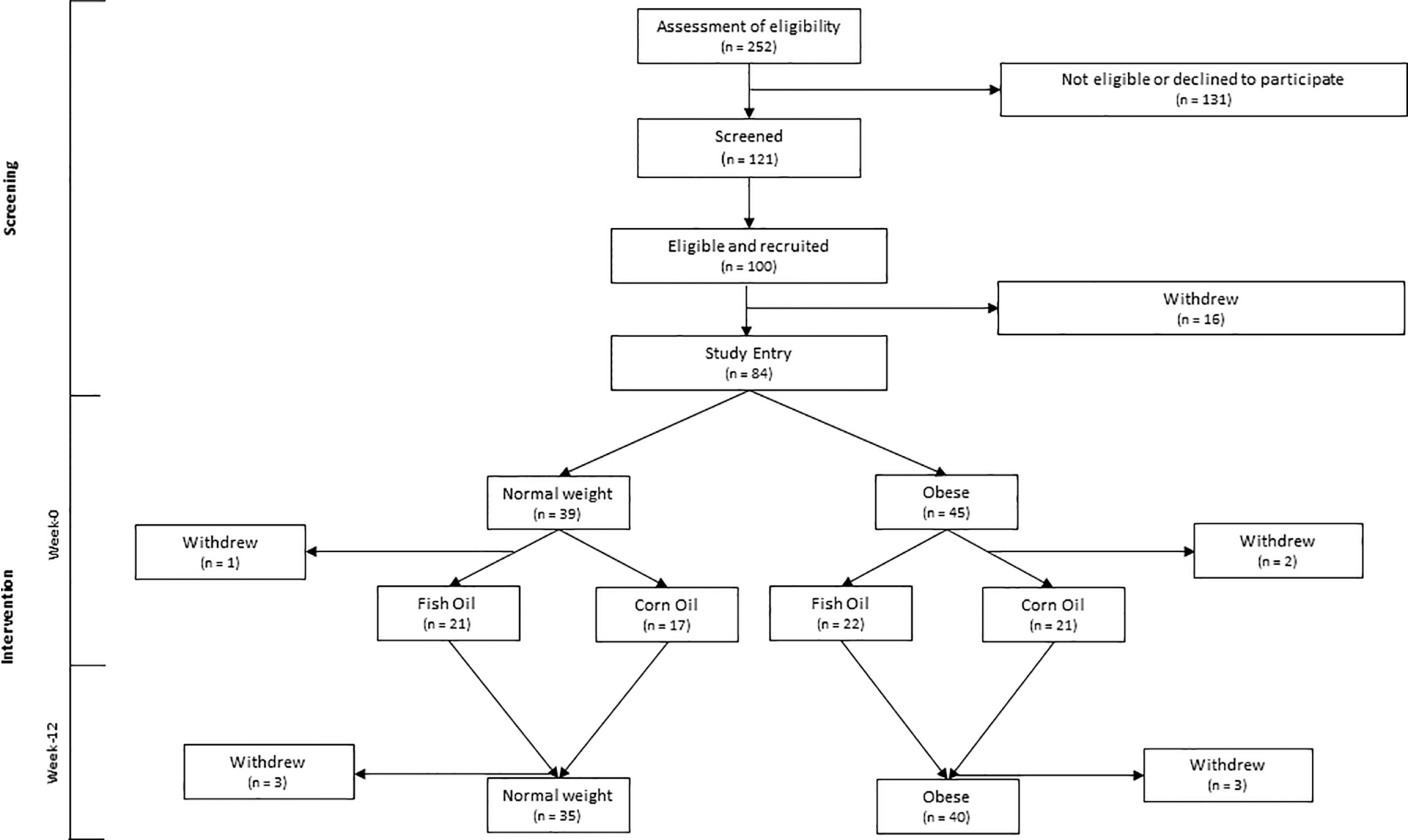
CONSORT diagram of participant inclusion and flow through the study.

Fasted blood and an abdominal scWAT biopsy (~1 g) were collected at baseline (week-0) and following 12-week intervention (week-12) during which participants were randomised to consume either 3 g of a concentrated fish oil (EPAX6000; EPAX, Alesund, Norway providing 1.1 g EPA + 0.8 g DHA) or 3 g of corn oil (providing 1.65 g linoleic acid and 0.81 g oleic acid) per day (**Figure 1**). Blinding, randomization, and supplement packaging were completed by the Research Pharmacy at Southampton General Hospital, Southampton, United Kingdom, by individuals independent of the researchers involved in the study. Treatment group blinding was maintained until completion of statistical analysis of all data.

### Sample Preparation

Abdominal scWAT biopsies were collected by surgical removal under local anaesthetic (1% lidocaine) to provide ~1 g of intact tissue which was directly stored on ice. Tissue was divided into 5 x ~200 mg aliquots. scWAT designated for FA and lipid metabolite analyses was wrapped in foil, placed in cryovials and snap frozen in liquid nitrogen. scWAT designated for RNA analysis was stored in 4 mL of RNAlater (Sigma, St. Louis, Missouri, United States) and stored for 24 hours between 2-4°C and then at -20°C for longer storage.

### Blood Analyses

Plasma was prepared from ~5 mL of heparinised blood as previously described (30). Plasma TG, cholesterol, high density lipoprotein cholesterol (HDL-C), nonesterified fatty acid (NEFA), and glucose concentrations were measured using an iLAB 600 clinical chemistry analyzer and software (Instrumentation Laboratories, Bedford, Massachusetts) and enzyme-based kits (Wako, Osaka, Japan). Low density lipoprotein cholesterol (LDL-C) concentrations were estimated by using the Friedwald equation. Plasma insulin concentrations were measured by ELISA (Dako, Agilent, Santa Clara, California) and homeostasis model assessment 2 of insulin resistance (HOMA2-IR) was calculated as follows as previously described (30). Concentrations of interleukin (IL)-6 and adiponectin were determined from fasted plasma using luminex performance assays as previously described (30).

### Anthropometry

Height was measured using a Seca stadiometer (Seca, Hamburg, Germany), waist and hip circumference measurements were made using a tape measure, and weight and body composition measurements were made using digital impedance apparatus (TANITA BC-418) as previously described (36).

### Fatty Acid Composition

Lipids were extracted from frozen scWAT and fatty acid methyl esters (FAMEs) formed as previously described (36). FAMEs were separated by gas chromatography on a BPX-70 fused silica capillary column (30 m x 0.2 mm x 0.25 µm; manufactured by SGE) in a HP6890 gas chromatograph fitted with a flame ionisation detector. Instrument run conditions were as described elsewhere (36).

### Gene Expression

RNA was isolated from ~150 mg scWAT stored in RNA*later* ^®^ using the RNeasy lipid tissue mini kit™ (QIAGEN, Hilden, Germany) as previously described (30). Sequencing was performed on a Hiseq2000 platform with 5 samples per lane in a total of 8 lanes (SE50) with a total of 20 million reads. RNA-Seq reads were aligned to the hg38.0 reference genome using TopHat (open source, Johns Hopkins University, Center for Computational Biology, Baltimore, United States) and a read count table produced using HTSeq (open source, Huber group, Heidelberg, Germany). The read counts were filtered, and normalised CPM were used to evaluate gene expression as previously described (30). The results from this subset were validated *via* qRT-PCR of RNA extracted from the whole cohort as previously described (30), and showed the subset to be representative of the whole cohort. Gene ontology functional annotation was performed using the Database for Annotation, Visualization and Integrated Discovery (DAVID) (37, 38). Gene set enrichment analysis was performed using GSEA (39, 40). Ingenuity pathway analysis (Qiagen, Hilden, Germany) was run to identify pathways and networks enriched amongst the differentially expressed genes.

### Histochemical Analyses

scWAT was fixed in 4 mL of 10% neutral buffered formalin and stored at room temperature and then embedded in paraffin wax. 4 µM sections were cut using a Leica RM2125 RTS microtome (Leica Biosystems, Wetzlar, Germany) and dried onto glass slides at 37°C overnight. A sub-set of 20 paired pre- and post-intervention samples (10 normal weight, 10 obese, including the 20 samples analysed for RNA-Sequencing) were selected for H&E staining, Picro Sirius red staining, and CD68 staining.

### Statistical Analysis

Sample size was calculated considering the typical distribution and expected response of circulating cytokines, not reported here (a 20% decrease following LC n-3 PUFA intervention) and participant drop out of 20%. A sample size of 25 participants per group (BMI and treatment subgroup) was determined to be able to detect changes in circulating cytokines at > 80% power and a 5% level of significance with consideration for 20% loss. No formal power calculation was performed specifically for the outcomes described herein. Not all data were normally distributed, and non-normal data could not be corrected with log_10_ transformation. Therefore, appropriate non-parametric tests were performed, and all data are displayed as median and interquartile range (IQR). 170 genes that were differentially expressed in individuals living with obesity by at least a 2 FC were selected for analysis using IPA.

## Results

### Participant Characteristics

Individuals living with obesity had significantly greater BMI, waist circumference, hip circumference, % body fat, body fat mass (kg), and higher blood concentrations of TG, total cholesterol, LDL-C, glucose, and insulin, in comparison to normal weight individuals (**Table 1**). Individuals living with obesity also had higher concentrations of IL-6 and leptin in comparison to normal weight individuals allocated to the corn oil intervention group. Individuals living with obesity also had lower adiponectin concentrations than normal weight individuals allocated to the corn oil intervention group (**Table 1**). Individuals living with obesity had a significantly higher average HOMA2-IR score; however, this was still within the ‘normal range’ (HOMA-IR < 1.95). As no individuals with diagnosed metabolic or inflammatory complications were recruited, and obese individuals did not exhibit clinical hypertriglyceridemia and had HOMA2-IR scores within the normal range, the obese cohort were defined as currently living with obesity in which metabolic syndrome is yet to manifest.

**Table 1:** Anthropometric and metabolic characteristics in normal weight and individuals living with obesity.

	^1^Normal weight - Fish oil	^1^Obese - Fish oil	^2^ *P*	^1^Normal weight - Corn oil	^1^Obese - Corn oil	^3^ *P*	Normal range
Age (years)	30.89 ± 15.26	47.43 ± 11.72	≤ 0.001	32.99 ± 15.22	40.79 ± 11.85	0.094	
Sex M/F	7/12	3/14	0.470	6/17	6/15	0.437	
BMI (kg/m^2^)	22.27 ± 1.64	33.97 ± 2.79	≤ 0.001	22.41 ± 1.77	35.60 ± 2.80	≤ 0.001	
Waist (cm)	74.95 ± 5.46	106.77 ± 10.85	≤ 0.001	76.48 ± 8.69	109.53 ± 12.85	≤ 0.001	
Hip (cm)	93.25 ± 3.95	115.43 ± 7.46	≤ 0.001	93.59 ± 5.83	119. 75 ± 8.42	≤ 0.001	
Body fat (%)	21.06 ± 8.64	41.13 ± 7.09	≤ 0.001	24.45 ± 6.06	41.61 ± 6.69	≤ 0.001	
Body fat mass (kg)	12.73 ± 4.63	38.94 ± 7.92	0.001	15.06 ± 4.02	41.91 ± 6.69	≤ 0.001	
Lean mass (kg)	50.00 ± 11.70	56.27 ± 7.92	0.109	47.18 ± 9.45	59.41 ± 12.06	0.002	
TG (mmol/L)	0.79 ± 0.33	1.44 ± 0.74	0.001	0.72 ± 0.24	1.29 ± 0.75	0.004	< 1.7 mmol/L
NEFAs (mmol/L)	0.45 ± 0.20	0.56 ± 0.15	0.073	0.63 ± 0.19	0.63 ± 0.26	0.952	< 0.72 mmol/L
TC (mmol/L)	4.59 ± 1.34	5.60 ± 0.90	0.009	4.38 ± 0.85	5.03 ± 0.92	0.039	< 5.0 mmol/L
HDL-C (mmol/L)	1.64 ± 0.46	1.55 ± 0.38	0.511	1.55 ± 0.27	1.40 ± 0.33	0.147	> 1.0 mmol/L
LDL-C (mmol/L)	2.79 ± 1.14	3.76 ± 0.85	0.005	2.69 ± 0.77	3.37 ± 0.82	0.016	< 3.0 mmol/L
Glucose (mmol/L)	4.74 ± 0.43	5.48 ± 0.87	0.002	4.71 ± 0.44	5.47 ± 0.98	0.005	<7.0 mmol/L
Insulin (µIU/L)	5.19 ± 2.26	11.50 ± 6.08	<0.001	5.82 ± 3.26	14.64 ± 7.08	≤ 0.001	2.6-24.9 µIU/L
^4^HOMA2-IR	0.71 ± 0.29	1.37 ± 0.55	<0.001	0.76 ± 0.42	1.91 ± 0.91	≤ 0.001	< 1.9
Interleukin-6 (pg/mL)	2.25 ± 1.44	2.43 ± 1.26	0.685	1.28 ± 0.91	2.42 ± 1.7	0.003	
Adiponectin (µg/mL)	7.92 ± 2.88	6.50 ± 4.31	0.235	10.73 ± 5.00	5.19 ± 1.81	0.001	
Leptin (ng/mL)	13.34 ± 11.71	44.88 ± 24.31	<0.001	12.15 ± 5.17	50.46 ± 25.35	≤ 0.001	

^1^Mean ± SD; ^2^P obtained from univariate general linear model analysis by comparison of obese and normal weight individuals allocated to fish oil intervention. ^3^P obtained from univariate general linear model analysis by comparison of obese and normal weight individuals allocated to corn oil intervention. ^4^ HOMA2-IR = HOMA2-IR = (((insulin mmol/L) x (glucose IU/L))/22.5) corrected for variations in hepatic and peripheral glucose resistance, increases in insulin secretion curve for plasma glucose concentrations above 10 mmol/L, and the contribution of circulation proinsulin). Modified from (36).

### Obesity Is Associated With a Specific scWAT Transcriptome Suggestive of Enhanced Expansion and Remodelling in Response to Inflammation

Sequencing of RNA extracted from human scWAT identified 789 genes differentially expressed by at least a two-fold change in individuals living with obesity in comparison to normal weight individuals (*P* and FDR ≤0.05). These 789 genes were further examined, and GO identified 170 genes that were enriched in tissue structure, remodelling, and expansion processes. The top enriched process was ECM organisation; other enriched processes included collagen organisation, angiogenesis and blood vessel remodelling, cell proliferation, and response to hypoxia (*P* < 0.001 **Table 2**). Top upregulated genes include epidermal growth factor like protein-6 (*EGFL6*), MMPs, collagens, integrins, and other genes associated with ECM structure. Top downregulated genes included other collagens and alpha-2 glycoprotein-1 (*AZGP1*) (*P* ≤ 0.003, **Table 3**).

**Table 2:** Top 25 enriched biological processes, determined by GO, in scWAT in individuals living with obesity.

Biological Process	Gene count	*P* value*
Extracellular matrix organization	58	3.20E-63
Collagen catabolic process	31	2.60E-39
Integrin-mediated signalling pathway	32	3.40E-34
Cell adhesion	52	4.90E-34
Collagen fibril organization	18	4.90E-21
Blood vessel remodelling	17	5.00E-21
Extracellular matrix disassembly	19	6.00E-17
Angiogenesis	27	6.20E-17
Endodermal cell differentiation	14	1.00E-16
Leukocyte migration	20	1.80E-14
Proteolysis	32	1.10E-12
Cell-matrix adhesion	16	1.10E-11
Bone resorption	10	1.50E-10
Negative regulation of angiogenesis	13	4.90E-10
Cell adhesion mediated by integrin	8	1.50E-08
Skeletal system development	15	5.00E-08
Negative regulation of cell proliferation	23	7.00E-08
Bone remodelling	7	8.70E-08
Positive regulation of cell migration	16	2.30E-07
Osteoclast differentiation	8	4.00E-07
Response to hypoxia	15	7.60E-07
Skin development	8	7.30E-06
Heterotypic cell-cell adhesion	7	9.90E-06
Positive regulation of bone resorption	6	1.60E-05
Osteoblast differentiation	11	1.90E-05

*P Value is Benjamini-Hochberg adjusted.

**Table 3:** The top differentially expressed genes associated with tissue structure and remodelling in scWAT in individuals living with obesity at study entry (week 0).

Gene	Fold Change	*P*
EGFL6	43.53	<0.001
MMP7	42.51	<0.001
MMP9	16.30	<0.001
DCSTAMP	15.02	<0.001
SPP1	11.86	<0.001
COL11A1	8.37	<0.001
COMP	5.24	0.003
TNC	5.01	<0.001
COL4A2-AS2	4.49	<0.001
LAMC3	4.39	<0.001
SIGLEC15	4.19	<0.001
ITGAD	3.73	<0.001
SPINK5	3.67	<0.001
ITGAX	3.65	<0.001
MMP12	3.61	<0.001
CCR2	3.51	<0.001
COL4A4	3.30	<0.001
COL9A3	-2.00	<0.001
COL6A6	-3.63	<0.001
AZGP1	-3.97	<0.001

Fold change and P values were obtained by comparison of individuals living with obesity and normal weight individuals in a general linear model likelihood ratio test in Edge R software.

**Figure 2:**
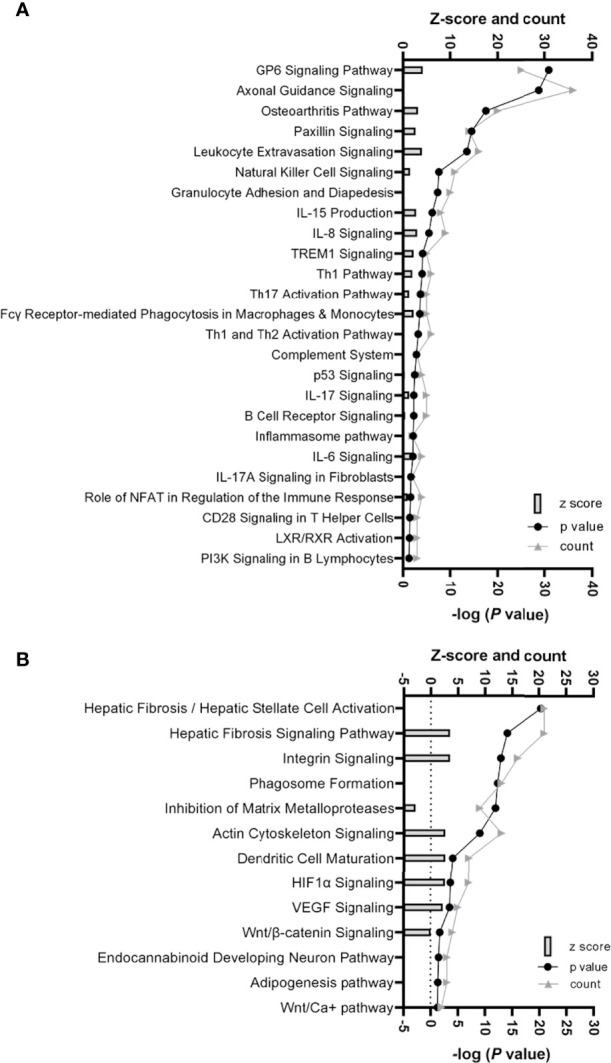
**(A)** Histogram of 25 significantly enriched canonical pathways involved in inflammation and immune response in scWAT in individuals living with obesity. **(B)** Histogram of significantly enriched canonical pathways involved in tissue remodelling and expansion in scWAT in individuals living with obesity. **(A, B)** The left y-axis represents the z-score (predicted score of activation/inhibition) and the number of genes counted for each of the representative canonical pathways, and the right y-axis represents -log (P-value), significance is deemed -log P> 1.3. Where z-score is not present, there was not enough evidence from current literature to determine if the altered gene expression is likely to result in activation or inhibition of a canonical pathway.

Ingenuity pathway analysis (IPA) (Qiagen, Hilden, Germany), which considers the differential expression, significance, counts per million (CPM), and false discovery rate (FDR) of the gene data, identified several canonical pathways to be upregulated in scWAT from individuals living with obesity. These processes fell into two major themes, upregulation of inflammatory and immune response processes and upregulation of tissue remodelling. We previously reported upregulation of the immune and inflammatory response in these individuals living with obesity when assessing the full gene set (30). In the current analysis, there is common overlap between inflammatory signalling and tissue expansion and remodelling. These enriched canonical pathways include upregulation of cytokine signalling, immune cell signalling and differentiation, and activation of inflammatory pathways such as the inflammasome pathway (*P* ≤ 0.05, **Figure 2A**). The enriched pathways involved in tissue remodelling include upregulation of hepatic fibrosis signalling, HIF-1α and vascular endothelial growth factor (VEGF) signalling, actin cytoskeleton signalling and dendritic cell maturation, Wnt/β-catenin signalling, and downregulation of inhibition of MMPs (*P* ≤ 0.05, **Figure 2B**).

**Figure 3:**
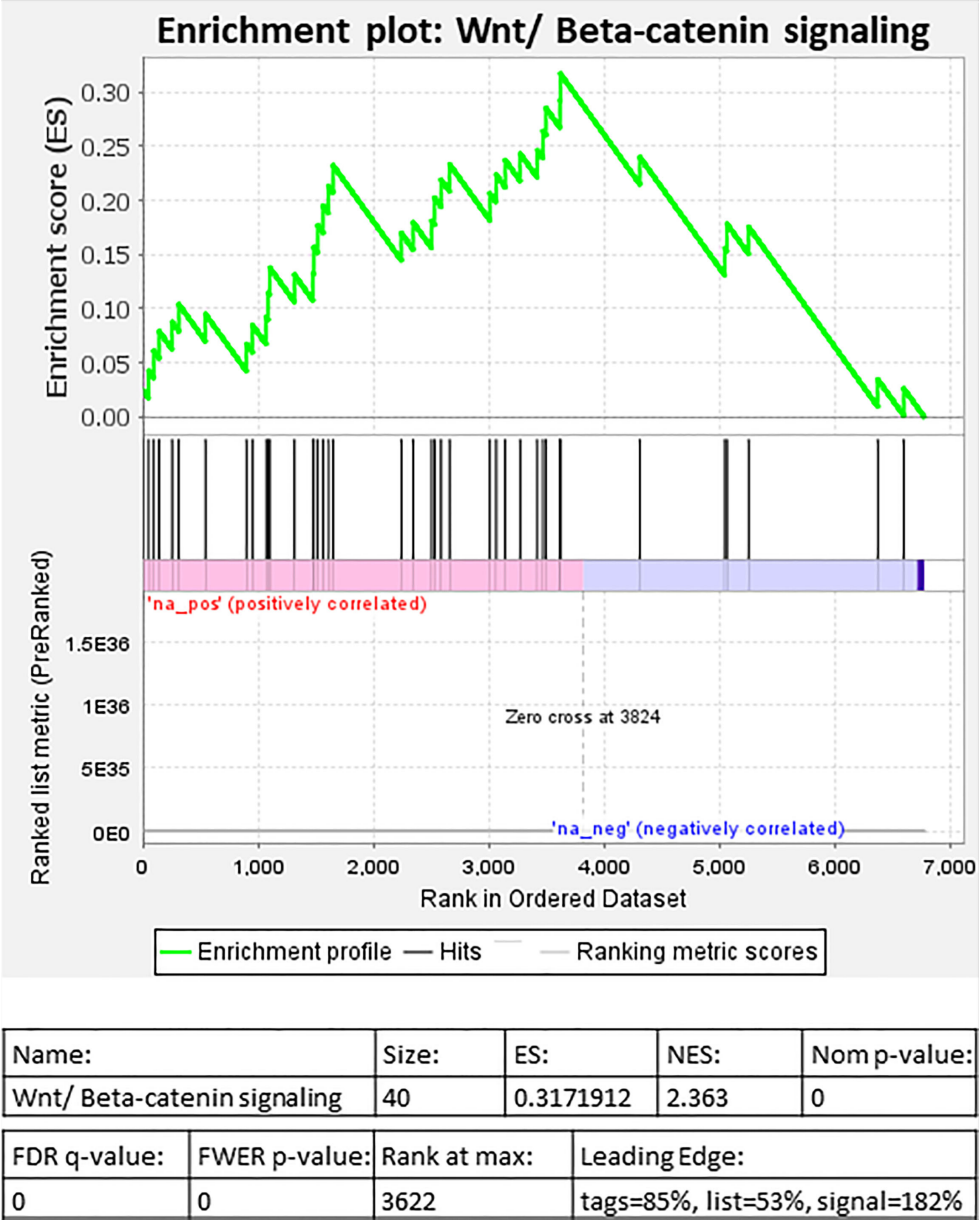
Gene set enrichment analysis (GSEA) of differentially expressed genes in individuals living with obesity at week-0: Enrichment plot for Wnt/β-Catenin signalling.

**Figure 4:**
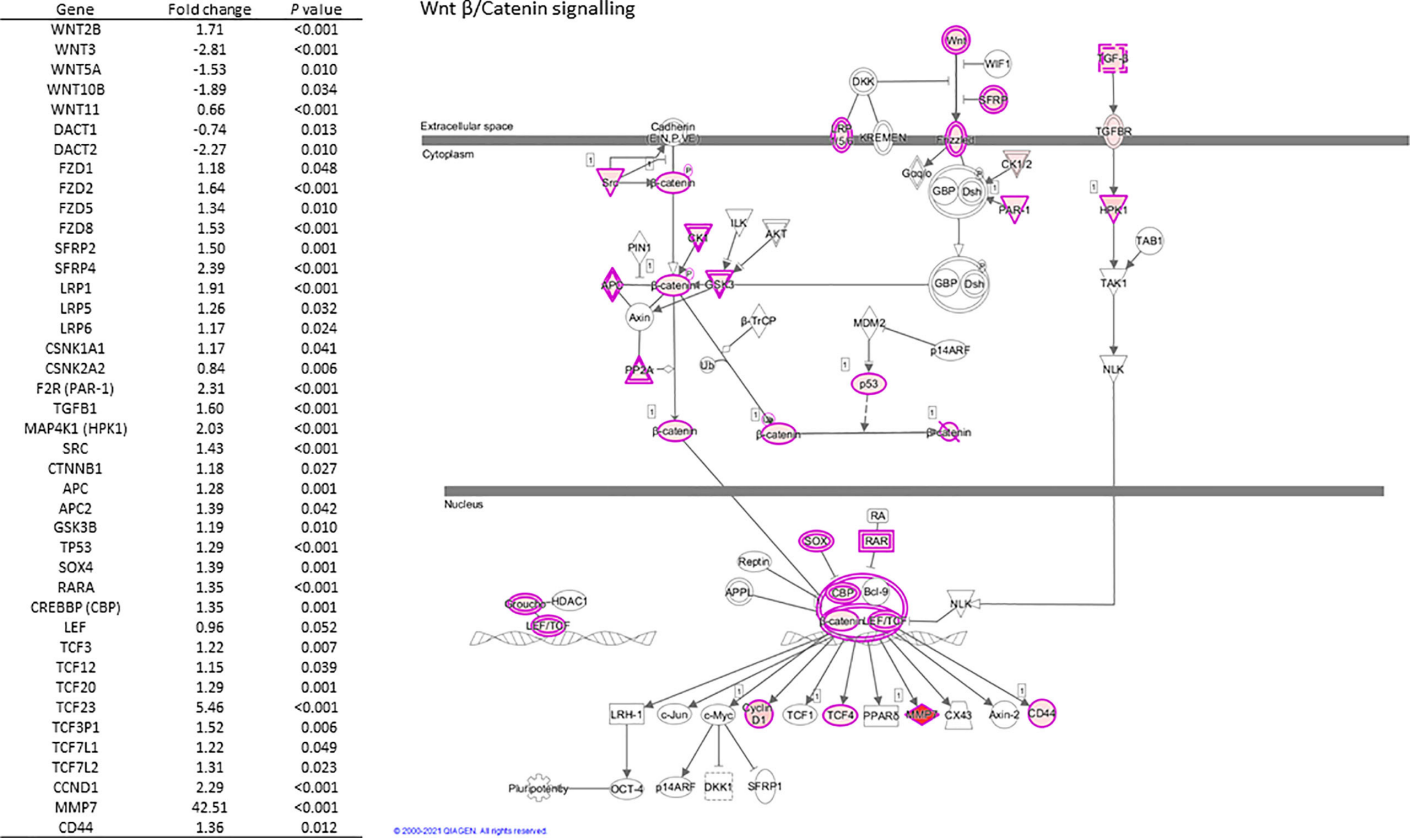
Regulation of scWATWnt/β-Catenin signalling and differential expression of pathway genes in individuals living with obesity at week-0.

Selected genes involved in several of the above pathways are detailed in **Table 3**. Higher expression of angiopoietin-2 (*ANGPT2*), *HIF-1α*, EGF like domain multiple 6 (*EGFL6*), several *MMP* genes, and growth/differentiation factor-15 (*GDF15*) was observed in individuals living with obesity (*P* ≤ 0.008, **Table 4**). There was significant enrichment of the Wnt/β-catenin signalling pathway; there was an overall positive enrichment of genes in this pathway but several of these are negative regulators of Wnt signalling (**Figure 3**). In addition, there was negative enrichment of several key Wnt signalling genes (**Figure 3**). This was concordant with individual gene expression and overall downregulation of Wnt/β-catenin signalling pathway (**Figure 4**). Several genes associated with the Wnt/β-catenin signalling pathway involved in adipogenesis and lipogenesis, were differentially regulated in individuals living with obesity (**Figure 2B**). Of note, there was lower expression of *DACT-2*, Wnt family member-3a (*WNT3A*), Wnt family member-5a (*WNT5A*), Wnt family member-10B (*WNT10B*) (*P* ≤ 0.033, **Table 4** and **Figure 4**), negative regulators of Wnt signalling, secreted frizzled related proteins *SFRP2* and *SFRP4* (*P* ≤ 0.001, **Figure 4**), and greater expression of Wnt receptors, frizzleds *FZD1*, *FZD2*, *FZD5* and *FZD8* (*P* = ≤ 0.048, **Figure 4**), co-receptors required for Wnt signalling, lipoprotein receptor-related proteins (LRPs) *LRP1*, *LRP5* and *LRP6* (*P* ≤ 0.032, **Figure 4**), and greater expression of T cell factor/lymphoid enhancer factors *TCF7L1* and *TCF7L2* (*P* ≤ 0.049, **Figure 4**) which repress target gene expression when Wnt signals are absent.

**Table 4:** Fold change of genes involved in expansion (hypertrophy and hyperplasia) and remodelling in scWAT in individuals living with obesity at study entry (week 0).

Gene	Fold Change	*P*
ANGPT2	1.45	0.008
DACT1	-0.74	0.884
DACT2	-2.27	0.010
EGFL6	43.41	<0.001
FZD2	1.64	<0.001
FZD1	1.18	0.048
FZD5	1.34	0.010
FZD8	1.53	<0.001
GDF15	2.64	<0.001
HIF1α	1.28	<0.001
LEP	2.08	<0.001
MMP7	42.52	<0.001
MMP9	16.22	<0.001
MMP12	3.61	<0.001
MMP24	2.11	<0.001
TCF7L2	1.31	0.020
VEGF	-1.56	<0.001
WNT3A	-2.81	<0.001
WNT5A	-1.53	0.010
WNT10B	-1.89	0.033

Fold change and P values were obtained by comparison of individuals living with obesity and normal weight individuals in a general linear model likelihood ratio test in Edge R software.

These data suggest obesity in which metabolic complications are yet to manifest is associated with a specific adipose transcriptome profile indicative of enhanced tissue remodelling and expansion in response to obesity-associated tissue inflammation and upregulation of immune response processes. In addition, we identify the dysregulation of the scWAT Wnt signalling pathway in obesity.

### Obesity Is Associated With scWAT Hypertrophy and Accumulation of Macrophages but Not Fibrosis

**Figure 5:**
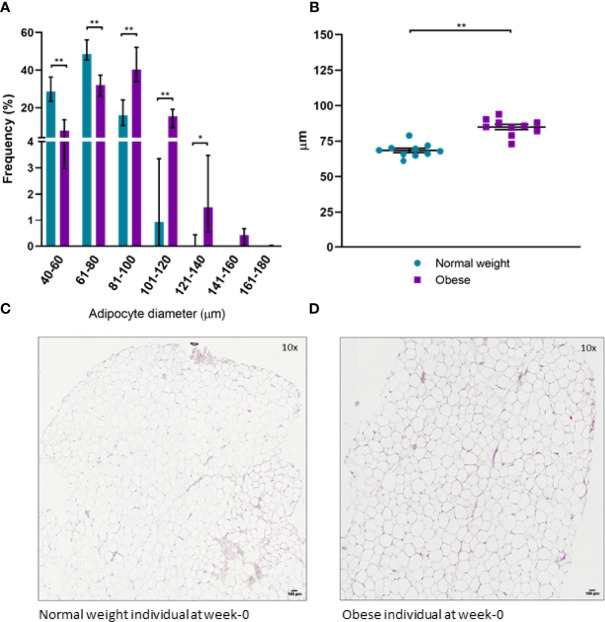
**(A)** Distribution of adipocyte size in scWAT in normal weight and individuals living with obesity at week-0; **(B)** Average adipocyte diameter in normal weight and individuals living with obesity at week-0; **(C)** H&E stained section of scWAT at 10 x magnification from a normal weight individual at week-0; **(D)** H&E stained section of scWAT at 10 x magnification from an individual living with obesity at week-0. **P < 0.001, *P < 0.050.

**Figure 6:**
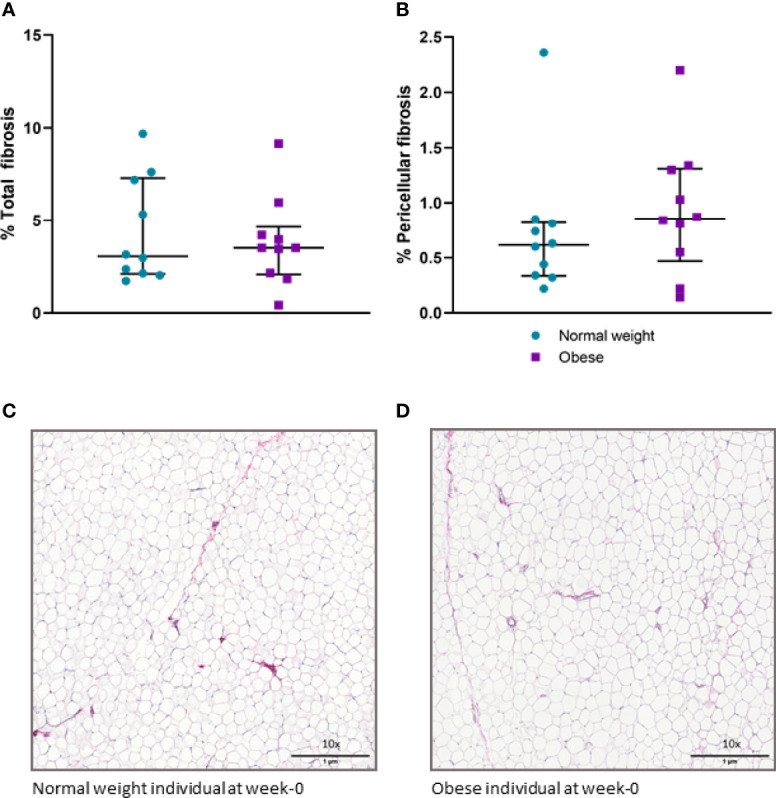
**(A)** Percentage of total fibrosis in scWAT in normal weight and individuals living with obesity at week-0; **(B)** Percentage of pericellular fibrosis in scWAT in normal weight and individuals living with obesity at week-0; **(C)** Picro Sirius red stained collagen in a section of scWAT at 10 x magnification from a normal weight individual at week-0; **(D)** Picro Sirius red stained collagen in a section of scWAT at 10 x magnification from an individual living with obesity at week-0.

**Figure 7:**
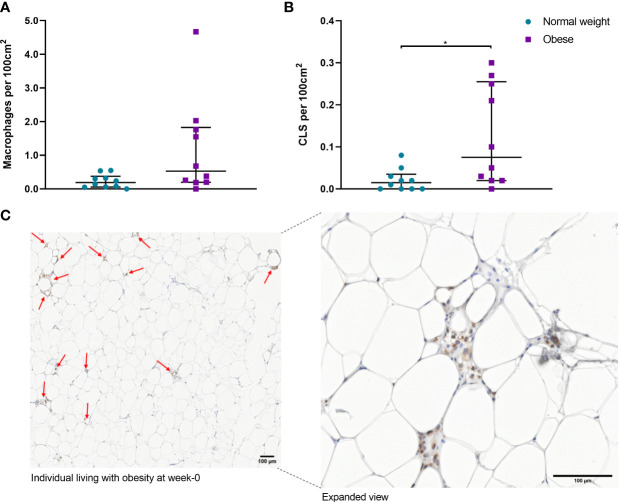
**(A)** Number of macrophages per 100cm^2^ of scWAT from normal weight and individuals living with obesity at week-0; **(B)** Number of CLS per 100 cm^2^ of scWAT from normal weight and individuals living with obesity at week-0; **(C)** CD68 stained macrophages in a section of scWAT at 20 x magnification from an individual living with obesity at week-0. *P = 0.023

Histochemical staining of scWAT revealed individuals living with obesity exhibit tissue hypertrophy in which the average adipocyte size was larger, in addition to a greater number of large, very large, and extra-large adipocytes in comparison to normal weight individuals (*P* ≤ 0.050, **Figures 5**, **6**). There was a greater number of macrophages accumulating in crown like structures (CLS) (*P* = 0.023) (defined as 3 or more macrophages aggregating around a single adipocyte) and a trend for higher numbers of macrophages in general (*P* = 0.063) in the scWAT of individuals living with obesity in comparison to normal weight individuals (**Figure 7**). The higher number of CLS present in scWAT of individuals living with obesity may reflect a higher proportion of pro-inflammatory M1 macrophages. The number of CLS per 100 cm^2^ of scWAT was positively correlated with BMI and body fat (kg) (ρ = 0.192, *P* = 0.023 and ρ = 0.219, *P* = 0.029 respectively) and number of macrophages per 100 cm^2^ of scWAT was positively correlated with body fat (kg) (ρ = 0.362, *P* = 0.041). However, despite being significant, these correlations are not particularly strong and do not indicate BMI or body fat (kg) to be a clear predictor of macrophage or CLS numbers. There were no significant correlations with insulin or HOMA2-IR (data not shown). There is variation in CLS number in obesity that is not explained by variation in blood lipid or metabolic parameters but may be due to variation in adipokines and cytokines. The number of CLS per 100 cm^2^ of scWAT was positively correlated with circulating IL-6 (*ρ*= 0.491, *P* = 0.028) and negatively correlated with circulating adiponectin concentrations (ρ = -0.477, *P* = 0.028). In addition, adipocyte size was positively correlated with IL-6 (ρ = 0.499, *P* = 0.025) and both adipocyte size and pericellular fibrosis were negatively correlated with adiponectin (ρ = -0.597, *P* = 0.005, and ρ = -0.617, *P* = 0.004, respectively). Pericellular fibrosis was also positively correlated with HOMA2-IR (ρ = 0.493, *P* = 0.027) but the level of this fibrosis was not altered in obesity suggesting this may be dependent on insulin sensitivity and may be more prevalent in the later stages of obesity coinciding with manifestation of metabolic syndrome.

**Figure 8:**
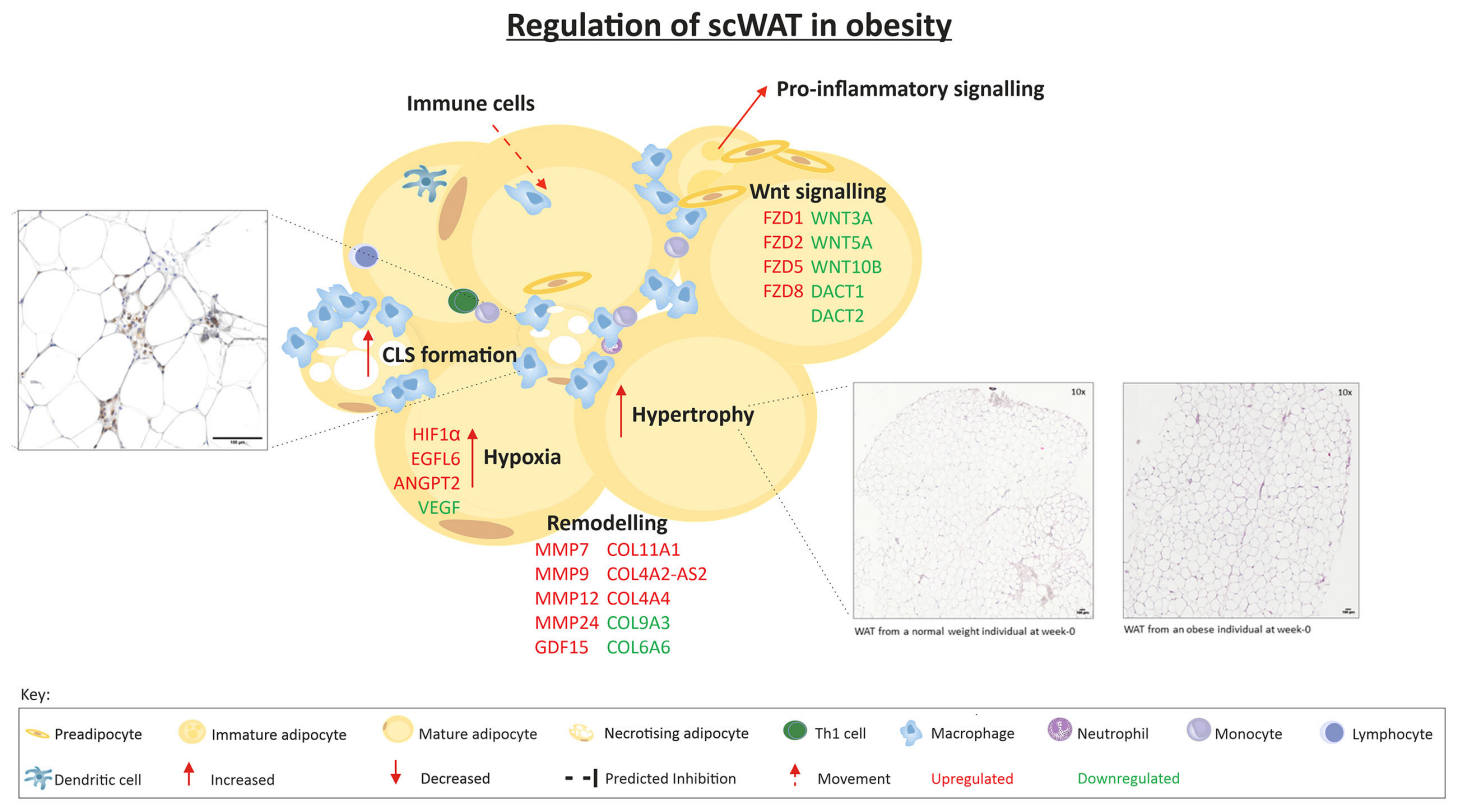
The regulation of scWAT in obesity in which metabolic complications are yet to manifest. Indication of upregulation or downregulation of mRNA expression is the expression in individuals living with obesity in comparison to that of normal weight individuals at week-0.

A summary of the regulation of scWAT in obesity is depicted in **Figure 8**.

### Chronic Supplementation With LC n-3 PUFA Modulates Expression of scWAT Genes but Does Not Alter Tissue Morphology

**Figure 9:**
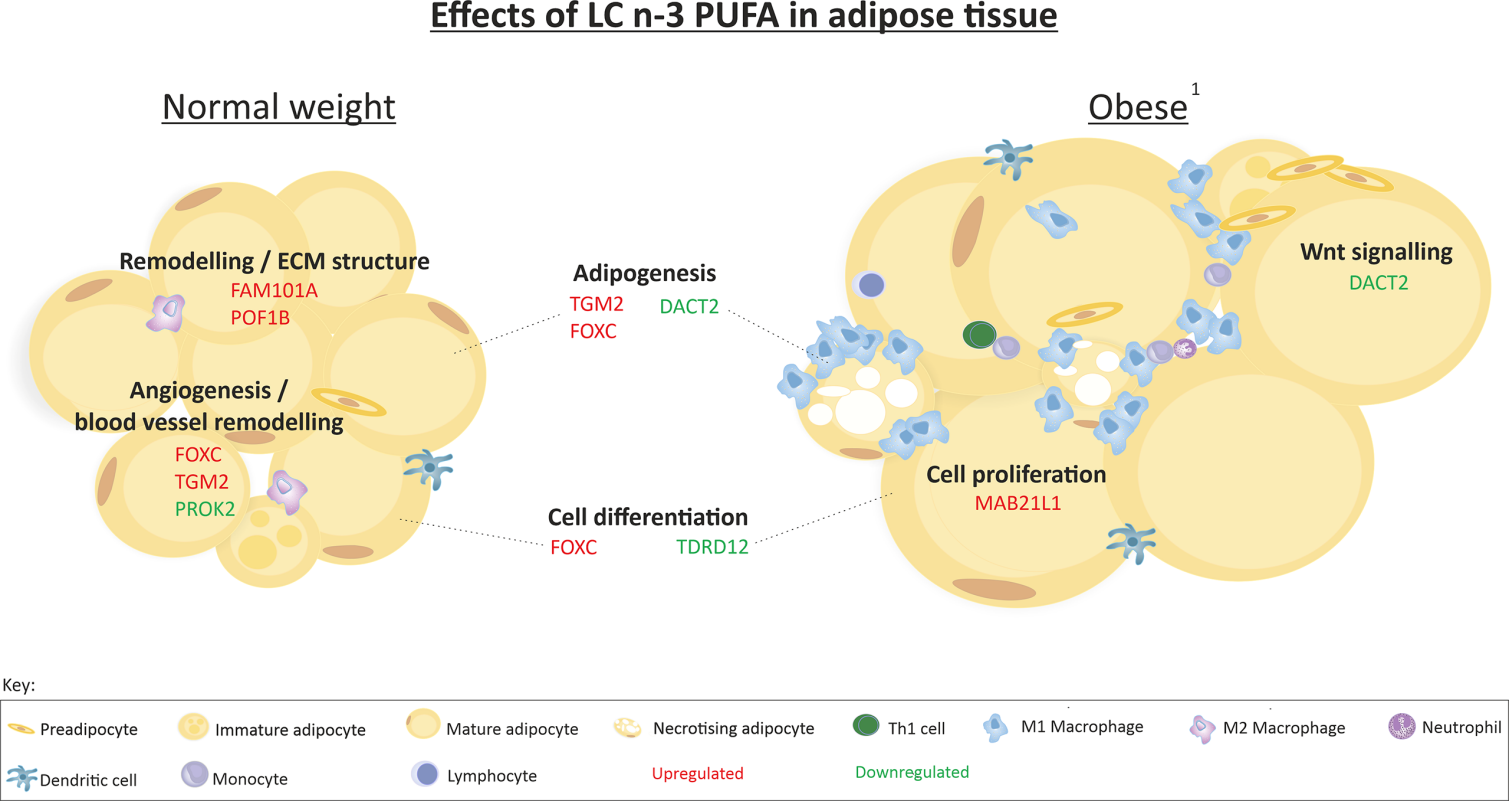
Summary of the effects of 12-week LC n-3 PUFA intervention on scWAT in normal weight individuals and individuals living with obesity. ^1^ Obesity in which metabolic complications are yet to manifest. Indication of upregulation or downregulation of mRNA expression is the expression at week-12 in comparison to week-0 in each group of individuals.

We previously reported that 12-weeks of LC n-3 PUFAs increased scWAT proportions of EPA, DPA, and DHA to a similar extent in both BMI groups but this was only significant in normal weight individuals (36). EPA, DPA and DHA also increased in erythrocytes following 12-week LC n-3 PUFA intervention in both normal weight individuals and those living with obesity (**Table 5**). 12-week EPA+DHA significantly modulated the expression of several genes involved in tissue remodelling and expansion processes (**Table 6**). These genes are associated with the upregulation of blood vessel remodelling, actin filament binding, cell differentiation, and apoptotic cell clearance in normal weight individuals, and with anatomical structure morphogenesis and the negative regulation of cell proliferation in individuals living with obesity. In addition, LC n-3 PUFAs downregulated genes associated with angiogenesis, inflammatory response and circadian rhythm in normal weight individuals, and downregulated genes associated with cell differentiation, negative regulation of cell adhesion, and Wnt signalling in individuals living with obesity. A summary of the effects of LC n-3 PUFAs on the regulation of scWAT in normal weight individuals and individuals living with obesity in which metabolic complications are yet to manifest is depicted in **Figure 9**.

**Table 5:** Erythrocyte fatty acids at study entry and following 12-week intervention with LC n-3 PUFAs or corn oil in normal weight and individuals living with obesity.

Week - 0 Fish oil				Week - 12 Fish oil				
Fatty acid	Normal weight^1^	Obese^1^	*P^2^*	Normal weight^1^	*P^3^*	Obese^1^	*P^3^*	*P^4^*
14:0	0.55 (0.41, 0.63)	0.49 (0.45, 0.61)	0.935	0.50 (0.47, 0.62)	0.648	0.50 (0.45, 0.64)	0.940	0.787
16:0	24.79 (24.04, 25.63)	25.26 (24.52, 26.49)	0.291	24.76 (24.06, 25.84)	0.242	25.18 (23.57, 25.90)	0.037	0.957
16:1n-7	0.77 (0.58, 1.05)	1.03 (0.84, 1.21)	0.035	0.77 (0.61, 0.99)	0.116	0.92 (0.72, 1.18)	0.086	0.110
18:0	13.22 (12.24, 13.84)	12.71 (12.14, 13.48)	0.482	12.93 (11.93, 13.52)	0.055	12.72 12.19, 13.46)	0.370	0.685
18:1n-9	17.96 (16.79, 18.45)	17.72 (16.90, 18.95)	0.646	17.12 (16.54, 17.79)	<0.001	17.01 (16.63, 17.68)	0.002	0.808
18:1n-7	1.36 (1.27, 1.52)	1.30 (1.16, 1.38)	0.070	1.38 (1.30, 1.44)	0.893	1.29 (1.21, 1.37)	0.654	0.058
18:2n-6	15.80 (14.93, 18.37)	15.26 (13.48, 16.29)	0.045	15.14 (14.62, 15.87)	0.007	15.52 (12.98, 16.02)	0.351	0.766
18:3n-6	0.18 (0.15, 0.20)	0.19 (0.13, 0.26)	0.685	0.13 (0.09, 0.17)	<0.001	0.16 (0.12, 0.22)	0.040	0.079
18:3n-3	0.33 (0.30, 0.45)	0.36 (0.35, 0.46)	0.137	0.35 (0.29, 0.43)	0.819	0.45 (0.33, 0.55)	0.279	0.045
20:0	0.11 (0.09, 0.15)	0.10 (0.08, 0.13)	0.105	0.10 (0.09, 0.14)	0.519	0.10 (0.08, 0.13)	0.681	0.317
20:1n-9	0.32 (0.27, 0.40)	0.30 (0.25, 0.42)	0.646	0.28 (0.24, 0.36)	0.015	0.26 (0.21, 0.31)	0.062	0.234
20:2n-6	0.27 (0.25, 0.29)	0.26 (0.22, 0.28)	0.117	0.25 (0.22, 0.26)	<0.001	0.22 (0.20, 0.26)	0.019	0.130
20:3n-6	1.85 (1.50, 2.22)	2.18 (1.88, 2.34)	0.040	1.33 (1.09, 1.61)	<0.001	1.75 (1.53, 1.81)	<0.001	0.002
20:4n-6	14.05 (13.45, 15.14)	14.20 (13.49, 14.70)	0.850	11.69 (10.83, 12.97)	<0.001	12.33 (11.72, 13.10)	<0.001	0.387
20:4n-3	0.07 (0.06, 0.09)	0.06 (0.06, 0.11)	0.892	0.06 (0.05, 0.07)	0.056	0.06 (0.06, 0.09)	0.100	0.482
20:5n-3	0.83 (0.68, 1.13)	1.15 (0.96, 1.24)	0.007	3.18 (2.71, 3.95)	<0.001	3.01 (2.60, 3.27)	<0.001	0.194
22:5n-3	2.53 (1.85, 2.68)	2.58 (2.12, 2.93)	0.213	3.22 (2.90, 3.44)	<0.001	3.09 (2.71, 3.42)	<0.001	0.317
22:6n-3	4.19 (3.63, 5.18)	4.63 (3.43, 5.55)	0.465	6.13 (5.79, 6.42)	<0.001	6.15 (5.51, 6.63)	<0.001	0.829
**Week - 0 Corn oil**				**Week - 12 Corn oil**				
**Fatty acid**	**Normal weight^1^**	**Obese^1^**	***P^2^***	**Normal weight^1^**	***P^5^***	**Obese^1^**	***P^5^***	***P^6^***
14:0	0.53 (0.44, 0.65)	0.54 (0.48, 0.70)	0.386	0.53 (0.48, 0.62)	0.589	0.53 (0.47, 0.68)	0.391	0.947
16:0	24.43 (23.90, 24.86)	25.03 (24.34, 25.79)	0.057	23.99 (23.21, 25.40)	0.116	24.95 (24.48, 25.44)	0.247	0.083
16:1n-7	0.80 (0.65, 0.94)	091 (0.72, 1.37)	0.117	0.79 (0.50, 1.03)	0.793	1.02 (0.82, 1.25)	0.478	0.026
18:0	13.28 (11.99, 13.94)	12.87 (12.13, 13.31)	0.205	13.25 (12.47, 13.91)	0.987	12.79 (12.27, 13.38)	0.737	0.243
18:1n-9	17.65 (17.08, 18.34)	18.41 (17.07, 19.00)	0.405	17.91 (16.63, 18.02)	0.232	18.17 (17.35, 18.83)	0.627	0.162
18:1n-7	1.37 (1.26, 1.52)	1.29 (1.20, 1.50)	0.505	1.36 (1.18, 1.44)	0.080	1.30 (1.22, 1.39)	0.654	0.894
18:2n-6	16.30 (15.21, 16.69)	16.02 (14.34, 16.46)	0.243	16.98 (16.10, 18.67)	0.008	15.77 (14.77, 17.02)	0.117	0.018
18:3n-6	0.20 (0.14, 0.22)	0.21 (0.18, 0.24)	0.182	0.19 (0.14, 0.25)	0.062	0.22 (0.18, 0.28)	0.191	0.334
18:3n-3	0.36 (0.30, 0.40)	0.43 (0.38, 0.51)	0.008	0.38 (0.32, 0.52)	0.731	0.44 (0.38, 0.55)	0.737	0.117
20:0	0.12 (0.10, 0.13)	0.11 (0.08, 0.15)	0.641	0.13 (0.10, 0.16)	0.471	0.10 (0.08, 0.14)	0.681	0.134
20:1n-9	0.34 (0.27, 0.39)	0.33 (0.26, 0.45)	0.947	0.32 (0.29, 0.48)	0.295	0.28 (0.25, 0.37)	0.156	0.152
20:2n-6	0.25 (0.23, 0.30)	0.27 (0.24, 0.31)	0.424	0.27 (0.25, 0.30)	0.544	0.27 0.23, 0.31)	0.940	0.790
20:3n-6	1.60 (1.38, 1.76)	2.21 (1.98, 2.38)	<0.001	1.63 (1.40, 1.79)	0.179	2.16 (1.99, 2.34)	0.526	0.001
20:4n-6	14.34 (12.77, 15.13)	13.36 (12.86, 14.91)	0.386	13.90 (12.92, 14.62)	0.085	13.47 (12.16, 14.67)	0.232	0.594
20:4n-3	0.07 (0.06, 0.08)	0.07 (0.05, 0.11)	0.920	0.07 (0.06, 0.09)	0.376	0.08 (0.06, 0.11)	0.823	0.841
20:5n-3	1.03 (0.88, 1.45)	1.07 (0.83, 1.15)	0.641	0.87 (0.74, 1.22)	0.422	1.04 (0.87, 1.18)	0.601	0.617
22:5n-3	2.46 (2.24, 2.76)	2.45 (2.22, 2.82)	0.973	2.34 (2.02, 2.56)	0.098	2.61 (2.22, 2.81)	0.654	0.117
22:6n-3	4.96 (4.07, 5.58)	3.95 (3.45, 4.98)	0.049	4.81 (3.74, 5.49)	0.342	4.09 (3.35, 4.95)	0.911	0.162

^1^Median (25^th^, 75^th^ percentile); ^2^P obtained from Mann-Whitney-U analysis by comparison of obese and normal weight at week-0, ^3^P obtained from Kruskal-Wallis analysis by comparison of week-0 and week-12 fish oil data within each BMI group, ^4^P obtained from Mann-Whitney-U analysis by comparison of obese and normal weight at week-12 post fish oil intervention, ^5^P obtained from Kruskal-Wallis analysis by comparison of week-0 and week-12 corn oil data within each BMI group, ^6^P obtained from Mann-Whitney-U analysis by comparison of obese and normal weight at week-12 post corn oil intervention.

**Table 6:** Remodelling associated genes significantly modulated by 12-weeks intervention with LC n-3 PUFAs in normal weight and individuals living with obesity.

		Normal weight individuals	
Gene ID	Full name	log_2_-Fold Change	^1^ *P* Value	FDR	GO: Biological Processes
FAM101A	Refilin-A	1.74	≤0.001	0.058	Regulation of chondrocyte development Actin filament binding
FOXC2	Forkhead box-C2	1.71	≤0.001	0.044	Blood vessel remodelling Cell differentiation
POF1B	Actin binding protein	1.69	≤0.001	0.019	Actin cytoskeleton organisation Epithelial cell morphogenesis
KIAA1644	Shisha like-1	1.56	≤0.001	0.015	Integral component of membrane
FBXO40	F box protein-40	1.45	≤0.001	0.089	Muscle cell differentiation Post translational protein modification
TGM2	Transglutamase-2	1.1	≤0.001	0.054	Apoptotic cell clearance Blood vessel remodelling
PROK2	Prokineticin-2	-1.87	≤0.001	0.024	Angiogenesis Inflammatory response Circadian rhythm
		**Individuals living with obesity**	
**Gene ID**	**Full name**	**^1^log_2_-Fold Change**	**^1^ *P* Value**	**FDR**	**GO: Biological Processes**
MAB21L1	MAB-21 like-1	1.06	≤0.001	0.002	Anatomical structure morphogenesis Negative regulation of cell proliferation
TDRD12	Tudor domain containing-12	-1.99	≤0.001	0.043	Cell differentiation DNA methylation in gamete generation
DACT2	Dishevelled binding antagonist of beta catenin-2	-1.25	≤0.001	0.080	Negative regulation of cell adhesion Regulation of Wnt signalling

^1^Log_2_ Fold change and P values were obtained by comparison of study entry (week-0) and post LC n-3 PUFA intervention (week-12) data of scWAT from individuals living with obesity in a general linear model likelihood ratio test in Edge R software.

## Discussion

### Dysregulation of Adipogenesis and Tissue Expansion in Obesity - The Role of Wnt Signalling

We report altered expression of several genes involved in scWAT adipogenesis and tissue expansion suggesting an upregulation of these processes in the early stages of obesity. We confirmed obesity-associated hypertrophy in which we observed significantly enlarged adipocytes in individuals living with obesity even before significant metabolic perturbations are obvious.

A group of proteins that have a role in controlling adipose growth and expansion in response to nutritional cues are encoded by the Wnt genes. Wnt signalling proteins bind to cell surface receptors composed of frizzleds and LRPs -5 and -6. This binding activates DACTs resulting in the inhibition of glycogen synthease 3-β kinase (GSK3) and of β-catenin phosphorylation (**Figure 4**) (41). Non-phosphorylated β-catenin translocates to the nucleus to regulate target gene expression. Wnt signalling proteins are anti-adipogenic and are inhibited by DACT proteins, so when there is high expression of DACTs, Wnt signalling is inhibited and adipogenesis occurs. Therefore, during WAT expansion, the expression of Wnts including *WNT3A* and *WNT10B* is decreased, often seen in conjunction with increased expression of DACT genes (16, 17). Our data support this and provide evidence for dysregulated mRNA expression of several key Wnt signalling components in human scWAT.

We observe downregulation of *WNT3A*, *WNT5A*, and *WNT10B* which is consistent with observation of enhanced TCF/LEF gene expression which represses target gene expression when Wnt signals are absent (42). Wnt effectors such as *TCF7L2* may be potential targets for therapeutic treatment of Wnt-related metabolic disease (43). *TCF7L2* mRNA expression is reported to be the strongest type-2 diabetes candidate gene and inactivation of *TCF7L2* leads to hepatic insulin resistance and decreased whole body glucose tolerance (43). In the current study, conversely, *TCF7L2* mRNA expression was negatively correlated with HOMA2-IR scores (ρ = -0.526, *P* = 0.017, data not shown). Non-canonical signalling of *WNT5A* is reported to contribute to obesity-induced inflammation and systemic insulin resistance in obese mice independent of tissue expansion (44). In contrast to previous reports of *WNT5A* positively correlating with IL-6 and insulin resistance (44, 45), data from the current study in which metabolic complications are yet to manifest, identifies negative correlations between *WNT5A* mRNA expression and HOMA2-IR (ρ = -0.480, *P* = 0.032) and plasma IL-6 concentrations (ρ = -0.445, *P* = 0.049). These data suggest that there is decreased *WNT5A* expression with declining metabolic health and increasing systemic inflammation. This is not consistent with previous reports but may reflect hijacking of the Wnt system by inflammatory cytokines such as IL-6 (46). Individuals living with obesity in the current study exhibited higher circulating concentrations of IL-6 and higher HOMA2-IR scores in conjunction with downregulation of scWAT *WNT5A* mRNA expression.

We also observed downregulated *DACT2* mRNA expression, which is conflicting with the inhibition of Wnt gene expression. *DACT1* has a well-defined role in inhibiting Wnt gene expression but the action of *DACT2* in human scWAT has yet to be defined (47). It may be that *DACT2* is involved in non-canonical Wnt signalling in human scWAT, or that there is dysregulation of this system in obesity which is consistent with reports that *DACT* expression increases only to the point where the expansion limit of the scWAT is reached (16). Obesity beyond this point results in loss of adipose function and in this scenario, loss of *DACT* expression in conjunction with loss of Wnt signalling is observed (16). In addition to adipogenesis, it has been reported that activation of Wnt/β-catenin signalling strongly inhibits the expression of collagen genes and stimulates the expression of matrix protease genes resulting in loss of matrix in chondrocytes and cartilage (48) so this signalling pathway may have a role in ECM regulation in obesity.

### Upregulation of Tissue Remodelling in Obesity – The Role of Hypoxia

We report altered expression of several genes involved in tissue remodelling as well as hypoxia and angiogenesis pathways in scWAT from individuals living with obesity. Data from the current study advances on evidence from a small cohort (n=25) of monozygotic BMI-discordant twins from the Finn-Twin study which exhibit differential expression of scWAT genes indicating upregulation of ECM remodelling associated genes including collagens, ECM glycoproteins, and proteogylcans (49–51). Data from the current study in a larger cohort of non-related adults with obesity, supports this limited evidence of upregulated collagen gene expression prior to metabolic complication but provides additional data describing lack of alteration to collagen deposition in the tissue itself despite altered regulation of a range of collagen genes.

We report dysregulation of collagen expression in individuals living with obesity observing both up- and down-regulated expression of individual collagens but we did not observe enhanced scWAT fibrosis in obesity. Collagen deposition in the tissue as measured by Picro Sirius red staining was positively correlated with insulin sensitivity, indicating association between tissue remodelling and insulin resistance, suggesting fibrosis may occur in the later stages of obesity accompanied by metabolic complications. The expression of *GDF-15* mRNA was upregulated in individuals living with obesity; serum GDF-15 concentration has recently been reported as a predictor of liver fibrosis in patients with NAFLD and was involved in the association between insulin resistance and liver fibrosis (52). The expression of *GDF-15* was not significantly associated with scWAT fibrosis in the current study but was positively associated with HOMA2-IR and leptin concentrations (*P* = 0.005, ρ = 0.599 and *P* = 0.007, ρ = 0.580 respectively), and negatively associated with adiponectin concentrations (*P* = 0.002, ρ = 0.657) suggesting association with metabolic health in scWAT.

In addition to fibrosis signalling, upregulated genes were associated with HIF-1α signalling, VEGF signalling, actin cytoskeleton, integrin and MMP signalling. Of note, *MMP-7* and *MMP-9* were highly upregulated by 42.5 and 16.3 fold in obesity, respectively. MMP-7 hydrolyses human plasminogen which results in angiogenic factors promoting blood vessel formation (53). It also releases TNF-α from the cell surface which has a role in inflammatory signalling but the role of MMP-7 in human obesity has not been defined (53). The current study is the first to report upregulation of *MMP7* mRNA expression which contrasts with previous reports of decreased expression in murine obesity (54) and decreased circulating concentrations in human obesity (55). MMP-9 is secreted from pericytes, fibroblasts and macrophages and may be reflective of increased inflammatory macrophage presence in scWAT in obesity (56). These data suggest scWAT from individuals living with obesity exhibits signs of hypoxia and an attempt to reconnect the vasculature with upregulation of blood vessel formation and remodelling as well as ECM remodelling.

### Enhanced scWAT Inflammatory Macrophage Infiltration in Obesity – Hypertrophy, Inflammation and Insulin Resistance

In obesity with accompanied insulin resistance, there is interaction between the fibrotic state of the scWAT and immune cell infiltration, thought to be due to the cascade of events occurring from adipocyte hypertrophy and consequential fibrosis. Given these interactions, it is suggested that scWAT infiltration of macrophages is correlated with fibrosis and insulin resistance (57); however, data from the current study do not support this. Neither total nor pericellular fibrosis was correlated with number of macrophages, number of CLS in the tissue, or HOMA2-IR. However, both number of macrophages and CLS were positively correlated with IL-6, and number of CLS was negatively correlated with adiponectin which plays a role in insulin sensitivity. This highlights the complexity of the relationship between fibrosis, immune cell recruitment, and insulin sensitivity.

Adipose dysfunction may manifest as a higher frequency of hypertrophic adipocytes in association with stress signals, necrosis, deposition of ECM components, and immune cell recruitment attributed to the pathological enlargement of adipocytes. Destabilisation of the ECM may lead to a reduction in mechanical stress on the expanding adipocytes and environment, and inflammation including macrophage infiltration only persists at the later stages of adipose dysfunction in response to an increasingly fibrotic ECM.

We report scWAT hypertrophy and increased CLS formation independent of tissue fibrosis which may suggest the balance between ECM breakdown and deposition is maintained in obesity in which metabolic complications have not yet manifested. However, the current study observes associations between number of CLS and pro-inflammatory cytokines, adiponectin and HOMA2-IR scores suggesting a more pro-inflammatory environment is associated with insulin signalling even in the absence of enhanced fibrosis and metabolic syndrome.

### LC n-3 PUFA Modulation of scWAT Transcriptome: Adipogenesis, Morphology and Remodelling

LC n-3 PUFA intervention significantly modulated genes involved in adipogenesis (*DACT2, TGM2* and *FOXC2*); however, the biological effects of this are uncertain as there may be an increase in adipogenesis *via* enhanced Wnt signalling due to the downregulation of its inhibitor *DACT2* with LC n-3 PUFA intervention (16, 58–60). As discussed, the actions of *DACT2* in adipose tissue are not defined; furthermore, the dysregulation of Wnt signalling at study entry in individuals living with obesity may interfere with physiological *DACT2* signalling. These data pave the way to further understand this signalling pathway by identifying the alteration of *DACT2* in human obesity and its modulation by dietary fatty acids. Elucidation of the role of DACT2 in adipogenesis may further our understanding of non-canonical Wnt signalling in obesity and how modulation by LC n-3 PUFAs affects adipogenesis in scWAT.

In addition to modulation of Wnt signalling, LC n-3 PUFAs upregulated the expression of genes associated with negative regulation of cell proliferation and cytokine mediated signalling in normal weight individuals, suggesting inhibition of inflammation. However, in individuals living with obesity, LC n-3 PUFAs upregulated the expression of genes associated with promoting cell differentiation and blood vessel remodelling suggesting intervention with these lipids may help improve the scWAT environment under circumstances of excess lipid accumulation and ECM restriction.

LC n-3 PUFA intervention did not alter scWAT morphological parameters. Changes to adipocyte size were not expected as there was no fat loss (data not shown) following the intervention period. In contrast to previous reports in scWAT of non-diabetic individuals with impaired glucose tolerance (61) and in visceral adipose tissue (62), the current study did not observe a reduction in scWAT macrophages in response to LC n-3 PUFAs. Despite this, we previously reported the downregulation of inflammatory and immune signalling in both groups of individuals with LC n-3 PUFA intervention (30).

### Strength and Limitations

The current study has several strengths including its sample size, compliance to the intervention which was >90%, and the careful phenotyping of the individuals. We have shown that 12 weeks of 1.9 g of EPA + DHA daily was adequate to increase EPA and DHA in human scWAT and to alter transcriptome profiles in both normal weight individuals and individuals living with obesity and that this has a novel influence on Wnt signalling. This dose of EPA + DHA could be achieved amongst the general population by diet (e.g. several servings of fatty fish weekly) or a combination of diet and supplementation.

A limitation of this study is that the study may be underpowered. A target group size of 40 normal weight individuals and 40 individuals living with obesity was required for the study to be appropriately powered to detect changes in circulating cytokines (using IL-6 as the primary outcome) at > 80% power and a 5% level of significance. There was no formal power calculation for the outcomes described in the current paper, and so it may be possible that the number of scWAT samples analysed from normal weight individuals was a limitation for some of the outcomes reported herein.

## Conclusion

In summary, the current study provides novel evidence for an altered transcriptome profile and tissue morphology in scWAT in obesity prior to manifestation of metabolic syndrome indicative of tissue hypertrophy, hypoxia, inflammatory signalling and macrophage infiltration, and remodelling, and for altered responses to LC n-3 PUFA according to adiposity. We report correlations between HOMA2-IR and scWAT fibrosis and *WNT* mRNA expression, as well as correlations between circulating IL-6 concentrations, macrophage infiltration and *WNT* mRNA expression suggesting an important role for metabolic health in addition to obesity. We highlight the dysregulation of scWAT Wnt signalling in human obesity and provide novel insights into the dysregulation of both canonical and non-canonical signalling. In addition, we report for the first time modulation of *DACT2* mRNA expression by LC n-3 PUFAs which may have beneficial effects on dysregulated Wnt signalling in individuals living with obesity.

We provide novel evidence that scWAT is responsive to dietary manipulation with LC n-3 PUFAs and that potential beneficial changes to the tissue environment through the modulation of inflammatory and remodelling pathways can be achieved within 12-weeks. Furthermore, we provide novel evidence for altered responses to LC n-3 PUFAs in human obesity. Higher doses of LC n-3 PUFAs and/or longer duration of the intervention period may result in morphological changes such as a decrease in the number of CLS and adipocyte size, which would be beneficial to individuals living with obesity.

## Data Availability Statement

The datasets presented in this study can be found in online repositories. The names of the repository/repositories and accession number(s) can be found below: https://www.ncbi.nlm.nih.gov/geo/, GSE162653.

## Ethics Statement

The studies involving human participants were reviewed and approved by National Research Ethics Service South Central–Berkshire Research Ethics Committee (submission no. 11/SC/0384). The patients/participants provided their written informed consent to participate in this study.

## Author Contributions

HF: data curation, formal analysis, investigation, methodology, writing - original draft, and writing - review and editing. CC: formal analysis, project administration, and writing - review and editing. EM: conceptualisation and writing - review and editing. RA: formal analysis and writing - review and editing. PN: formal analysis, project administration, and writing - review and editing. CP-C: investigation, project administration, and writing - review and editing. EA: data curation and writing - review and editing. KL: data curation and writing - review and editing. PC: conceptualisation, funding acquisition, supervision, and writing - review and editing. All authors contributed to the article and approved the submitted version.

## Funding

European Commission, Seventh Framework Programme (Grant Number 244995).

## Conflict of Interest

PCC undertakes unpaid voluntary work as the current President of the Federation of European Nutrition Societies (FENS) and as Past President of ILSI Europe.

The remaining authors declare that the research was conducted in the absence of any commercial or financial relationships that could be construed as a potential conflict of interest.

## Publisher’s Note

All claims expressed in this article are solely those of the authors and do not necessarily represent those of their affiliated organizations, or those of the publisher, the editors and the reviewers. Any product that may be evaluated in this article, or claim that may be made by its manufacturer, is not guaranteed or endorsed by the publisher.

## Abbreviations

Dishevelled binding antagonist of beta catenin, (DACT, alias DAPPER1); triglyceride, (TG); Hypoxia inducible factor-1α, (HIF-1α); Extracellular matrix, (ECM); Subcutaneous WAT, (scWAT); Matrix metalloproteinases, (MMPs); Interleukin-6, (IL-6); Eicosapentaenoic acid, (EPA); Long chain omega-3 polyunsaturated fatty acids, (LC n-3 PUFAs); Peroxisome proliferator activated receptors, (PPARs); High density lipoprotein cholesterol, (HDL-C); Low density lipoprotein cholesterol, (LDL-C); Homeostasis model assessment 2 of insulin resistance, (HOMA2-IR); Fatty acid methyl esters, (FAMEs); RNA integrity, (RIN); Counts per million, (CPM); Interquartile range, (IQR); Ingenuity pathway analysis, (IPA); Vascular endothelial growth factor, (VEGF); EGF like domain multiple 6, (*EGFL6*); Growth/differentiation factor-15, (*GDF15*); Wnt family member-3A, (*WNT3A*); Wnt family member-10B, (*WNT10B*); Crown like structures, (CLS).
